# The Perfect Circle Technique Shows Poor Inter-rater Reliability in Measuring Anterior Glenoid Bone Loss on Magnetic Resonance Imaging

**DOI:** 10.1016/j.asmr.2024.100905

**Published:** 2024-02-03

**Authors:** Nata Parnes, Clare K. Green, Emily I. Wynkoop, Adam Goldman, Keith Fishbeck, Kyle J. Klahs, Robert H. Rolf, John P. Scanaliato

**Affiliations:** a Department of Orthopaedic Surgery and Rehabilitation, Carthage Area Hospital, Carthage, New York, U.S.A.; b Department of Orthopaedic Surgery and Rehabilitation, Claxton Hepburn Medical Center, Ogdensburg, New York, U.S.A.; c George Washington University School of Medicine, Washington, District of Columbia, U.S.A.; d Beacon Orthopaedics & Sports Medicine, Cincinnati, Ohio, U.S.A.; e Department of Orthopaedic Surgery, Texas Tech University Health Science Center, El Paso, Texas, U.S.A.; f Midwest Orthopaedics at Rush University Medical Center, Chicago, Illinois, U.S.A.

## Abstract

**Purpose:**

To evaluate the reliability of the perfect circle methodology for measurement of glenoid bone loss in patients with anterior glenohumeral instability.

**Methods:**

We performed a chart review of retrospectively collected patients who underwent isolated arthroscopic anterior labral repair between January 1 and June 30, 2021, using our institution’s electronic medical records. The inclusion criteria included isolated anterior shoulder instability with anterior labral repair and corroborated tears on magnetic resonance imaging. A total of 9 raters, either sports or shoulder and elbow fellowship-trained orthopaedic surgeons, each evaluated the affected shoulder magnetic resonance imaging scans twice, with a minimum of 2 weeks between measurements. Measurements followed the “perfect circle” technique and included projected anterior-to-posterior glenoid diameter, amount of posterior bone loss, and percentage of posterior bone loss. Intrarater reliability and inter-rater reliability were then determined by calculating intraclass correlation coefficients (ICCs).

**Results:**

Ten consecutive patients meeting the selection criteria were chosen for inclusion in this analysis. Average estimated bone loss for the cohort was 2.45 mm, and the mean estimated glenoid diameter of the involved shoulder was 28.82 mm. The average percentage of bone loss measured 8.54%. The ICC for interobserver reliability was 0.55 for the perfect circle diameter and 0.17 for the anterior bone loss measurement (poorly to moderately reliable). The ICC for intraobserver reliability was 0.69 for the perfect circle diameter and 0.71 for anterior bone loss (moderately reliable).

**Conclusions:**

The perfect circle technique for estimating anterior glenoid bone loss on magnetic resonance imaging was found to have moderate intrarater reliability; however, reliability between observers was found to be moderate to poor.

**Level of Evidence:**

Level IV, diagnostic case series.

Glenoid bone loss (GBL) is a common finding in the setting of anterior glenohumeral instability.^1^, ^2^, ^3^, ^4^ Measurable GBL may be noted in up to 40% of all patients who present with anterior instability and over 90% of patients with recurrent instability.^5^, ^6^, ^7^, ^8^ Assessing GBL is an important step in the preoperative planning for patients with instability because bone loss is a known risk factor for failure after soft-tissue stabilization procedures.^1^^,^^5^^,^^9^, ^10^, ^11^, ^12^ Glenoid defects greater than 25% have been associated with failure rates of up to 75% after Bankart repair, and these patients are often indicated for bony augmentation procedures.^13^ However, the minimally accepted threshold of anterior GBL for arthroscopic labral repair has not yet been established, and recent data suggest that even “subcritical” anterior GBL of 13.5% may portend inferior outcomes after soft-tissue stabilization.^10^

GBL is commonly assessed using a best-fit “perfect circle” method, first described by Sugaya et al.^14^ Although 3-dimensionally reconstructed computed tomography (CT) with humeral head subtraction is currently accepted as the gold standard for assessing glenoid morphology,^5^^,^^6^^,^^9^^,^^15^^,^^16^ these studies are costly to the patient and result in significant radiation exposure. Several studies have shown comparable measurements using magnetic resonance imaging (MRI), and many surgeons now routinely use MRI in their practices to assess GBL during preoperative planning.^17^^,^^18^ Although previous studies have attempted to validate the reliability of this methodologic technique, concern persists regarding the accuracy and validity of this method in the clinical setting. Specifically, when not given a specific image to measure—or when multiple assessors apply the perfect circle technique—is GBL reliably assessed?

The purpose of this study was to evaluate the reliability of the perfect circle methodology for measurement of GBL in patients with anterior glenohumeral instability. We hypothesized that our multi-surgeon study would show good inter-rater and intrarater reliability, supporting the use of the perfect circle technique for evaluating GBL on preoperative MRI.

## Methods

### Patient Selection

Patients who underwent isolated arthroscopic anterior labral repair at a single high-volume shoulder/elbow and sports practice between January 1 and June 30, 2021, were identified. The inclusion criteria included a history of an anterior glenohumeral dislocation, age younger than 50 years, and availability of a 3-T MRI scan of the affected shoulder.

Patients were excluded if they had a history of shoulder surgery or instability due to generalized hyperlaxity. Ten consecutive patients who met the inclusion criteria were selected for this analysis. A surgeon (N.P.) who did not participate in the validation experiment served as a local study lead and ensured that the patient identifiers remained blinded to the assessors.

### Technique

All MRI scans were performed with the same imaging protocol and the same 3-T MRI system (sequences included T1-weighted, T2-weighted, and T2 fat-suppressed sequences) and were acquired in the plane of the glenoid. Images were assessed by 9 attending orthopaedic surgeons trained in either sports or shoulder and elbow fellowships using the Medstrat Joints picture archiving and communication system (version 5.37.0; Downers Grove, IL). All participating surgeons routinely manage anterior glenohumeral instability in practice, with approximately 264 anterior instability procedures performed annually by the practice. All surgeons were familiar with, and routinely used, the aforementioned picture archiving and communication system.

**Fig 1:**
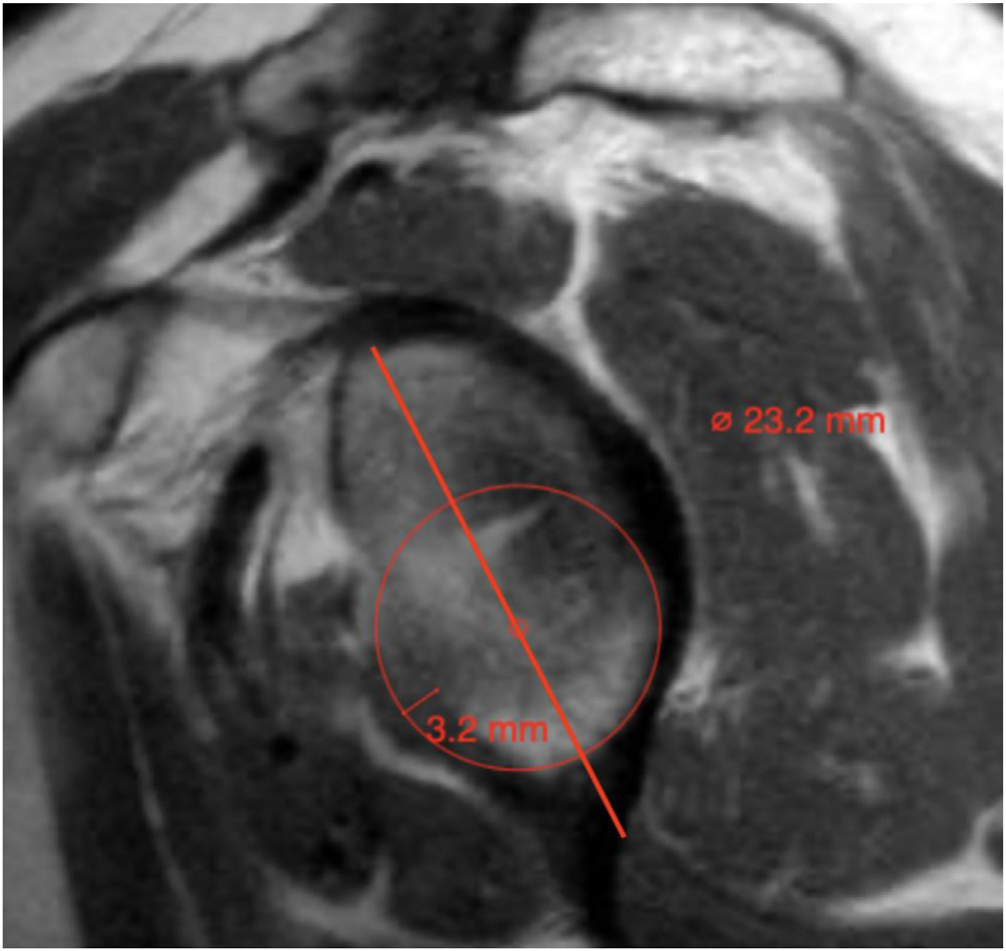
Sagittal view at glenoid face of left shoulder showing 3.2 mm of glenoid bone loss using perfect circle method on magnetic resonance imaging. (Ø, circle diameter.)

All surgeons were provided a technique guide outlining the steps for estimating anterior GBL using the perfect circle technique, as described by Sugaya et al.^14^ (Appendix Table 1). The measuring surgeons were not told from which series and image to obtain their measurements, and the best en face sagittal MRI scan of the glenoid, in the opinion of the measuring surgeon, was used. To obtain measurements, a line was first drawn along the long axis of the glenoid. A best-fit circle based on the inferior two-thirds of the glenoid was then approximated (Fig 1).

**Table 1:** Study Population Characteristics

	Mean ± SD (Range) or % (n)	Patient Data			
		Age, yr	Sex	Arm Dominance	No. of Dislocations
Age, yr	30 ± 10 (17-49)	—	—	—	—
Male sex	60 (6)	—	—	—	—
Dominant arm	50 (5)	—	—	—	—
Right shoulder	50 (5)	—	—	—	—
Patient No.					
1	—	49	F	Nondominant	4
2	—	30	F	Nondominant	2
3	—	42	M	Dominant	1
4	—	18	M	Dominant	1
5	—	25	F	Nondominant	1
6	—	29	M	Nondominant	2
7	—	27	M	Dominant	1
8	—	17	M	Dominant	1
9	—	39	M	Nondominant	3
10	—	23	F	Dominant	1

F, female; M, male; SD, standard deviation.

Total GBL and percentage of GBL were calculated using the following procedure: To calculate total GBL, a line was drawn along the long axis of the glenoid, spanning the perceived bone loss. The expected diameter was then calculated by drawing a line perpendicular to the long-axis line to create a line representing the diameter of the perfect circle. The percentage of bone loss was calculated based on these 2 lines in the standard fashion.

Each surgeon took 2 measurements for each patient, with a minimum of 2 weeks between measurements. After their measurements were completed, the site coordinator recorded the measured expected diameter, the GBL, the percentage of GBL, and the series and image number from which the measurements were obtained.

### Statistical Analysis

All statistical analyses were performed with RStudio (2023 release; Posit Software [PBC], Boston, MA). Inter-rater reliability was determined by calculating intraclass correlation coefficients (ICCs) based on average measurements from investigators. In addition, intrarater reliability was determined from separate sets of measurements from each investigator performed a minimum of 2 weeks apart. ICC values were evaluated based on the thresholds proposed by Koo and Li^19^: less than 0.5 represented poor reliability; 0.5 to 0.75, moderate reliability; 0.75 to 0.9, good reliability; and 0.9 or greater, excellent reliability.

## Results

Within the study period, 10 cases were chosen for inclusion in this analysis. The average age was 30 ± 10 years, and 60% of patients were male patients. The dominant arm was involved in 50% of cases, and 50% of cases involved the right shoulder. All patients were athletes, and the number of dislocations ranged from 1 to 4 dislocations (Table 1).

Average measured GBL for the patient cohort was 2.45 mm, and the mean glenoid diameter of the involved shoulder was 28.82 mm. The average percentage of GBL measured 8.54% (Table 2).

**Table 2:** Average Glenoid and Bone Loss Measurements by Patient

	Glenoid Diameter Using Perfect Circle Technique, mm	Bone Loss, mm	% Bone Loss
Patient 1	26.5 ± 2.3 (22.8-31.4)	4.2 ± 1.9 (0-8)	15.7 ± 6.5 (0-28.8)
Patient 2	26.1 ± 2.2 (21.7-29.1)	3.6 ± 2.0 (0-6.5)	13.6 ± 7.2 (0-22.8)
Patient 3	31.3 ± 1.3 (29.3-33.7)	1.8 ± 1.7 (0-6.9)	5.6 ± 5.4 (0-22.3)
Patient 4	27.8 ± 2.8 (21.7-34.3)	2.0 ± 1.4 (0-3.8)	7.1 ± 4.8 (0-12.6)
Patient 5	26.2 ± 2.0 (22.7-30.5)	1.3 ± 1.2 (0-3.0)	5.1 ± 4.6 (0-12.5)
Patient 6	28.0 ± 1.9 (25-32.2)	2.2 ± 1.3 (0-3.8)	7.8 ± 4.8 (0-14.1)
Patient 7	31.4 ± 1.7 (27.7-34.2)	2.4 ± 2.1 (0-7.5)	7.6 ± 6.6 (0-23.5)
Patient 8	30.6 ± 2.4 (26-35.5)	2.5 ± 1.2 (0-4.1)	8.1 ± 3.8 (0-13.4)
Patient 9	31.3 ± 2.3 (27.6-38.1)	2.5 ± 2.0 (0-6.8)	7.9 ± 6.3 (0-21.7)
Patient 10	29.0 ± 2.4 (25.7-34.6)	2.0 ± 2.2 (0-6.4)	6.9 ± 8.2 (0-24.1)
Average	28.8 ± 3.0	2.5 ± 1.9	8.5 ± 6.8

NOTE. Data are presented as mean ± standard deviation (range).

The ICC for interobserver reliability was 0.55 for the perfect circle diameter and 0.17 for the anterior GBL measurement. The ICC for intraobserver reliability was 0.69 for the perfect circle diameter and 0.71 for anterior GBL (Table 3).

**Table 3:** Inter-rater and Intrarater Reliability

	ICC	95% CI
Interobserver reliability		
Perfect circle diameter	0.552	0.302-0.820
Anterior bone loss	0.179	0.051-0.475
Intraobserver reliability		
Perfect circle diameter	0.690	0.57-0.78
Anterior bone loss	0.710	0.59-0.80

CI, confidence interval; ICC, intraclass correlation coefficient.

The average variance from the mean for all 3 measurements (glenoid diameter, bone loss, and percentage of bone loss) for each reviewer was calculated. Reviewer 4 was a slight outlier; this reviewer measured, on average, 2.6 mm off of the collective mean, which translated to a 9.4% bone loss difference compared with the other reviewers’ average measurements. Otherwise, the reviewers’ measurements of bone loss were within 6% of each other (Table 4). All measurements per reviewer are included in Appendix Table 1.

**Table 4:** Variance from Average Measurement per Reviewer

	Glenoid Diameter Using Perfect Circle Technique, mm	Bone Loss, mm	% Bone Loss
Reviewer 1	1.0 (0.1-3.5)	0.8 (0.5-1.8)	2.6 (0.2-5.6)
Reviewer 2	1.0 (0.1-3.3)	1.1 (0.2-3.1)	3.5 (0.2-11.0)
Reviewer 3	1.3 (0.5-2.3)	1.3 (0.3-2.6)	4.3 (1.1-7.1)
Reviewer 4	1.3 (0.2-2.85)	2.6 (0.9-5.0)	9.4 (3.1-16.8)
Reviewer 5	2.2 (0.7-4.6)	1.8 (0.8-3.6)	5.9 (2.3-13.6)
Reviewer 6	2.7 (0.3-5.9)	0.7 (0-2.0)	2.1 (0-7.2)
Reviewer 7	1.4 (0.3-3.1)	0.5 (0.1-1.4)	1.6 (0.1-4.7)
Reviewer 8	0.8 (0.3-2.5)	0.6 (0.1-1.5)	2.0 (0.3-4.9)
Reviewer 9	0.9 (0.2-2.1)	1.4 (0.2-3.4)	4.7 (1.0-12.4)

NOTE. Data are presented as mean (range).

## Discussion

The findings of this study suggest that for a single observer, the perfect circle technique is moderately reliable for assessing anterior GBL on MRI. However, between observers, reliability was found to be moderate to poor across 10 patients over 2 sets of measurements. Together, these findings contradict our hypothesis and suggest that the perfect circle methodology may represent a suboptimal technique for assessing anterior GBL with MRI.

GBL is a well-described consequence of acute instability events.^5^^,^^9^^,^^11^ A study by Dickens et al.^11^ showed that a single instability event results in a mean of 6.8% GBL with some patients with first-time instability events experiencing GBL of 13.5% or greater. Properly identifying and characterizing GBL is a critical step in the preoperative planning process for patients with anterior instability given that GBL is a known risk factor for failure after soft-tissue stabilization.^5^^,^^11^^,^^13^ It therefore follows that the validity of the perfect circle technique is an important aspect of one’s treatment algorithm because correct quantification of GBL allows for selection of the most appropriate surgical procedure. However, our findings suggest that despite following a standardized protocol for obtaining measurements, the interobserver reliability among 9 fellowship-trained orthopaedic surgeons for the perfect circle diameter and GBL is moderate to poor. This is of particular concern given the rising evidence that even subcritical GBL to the low teens may predispose patients to poor outcomes after isolated Bankart repair.^10^ Failure to recognize critical or subcritical GBL may lead surgeons to perform soft-tissue stabilization in cases in which bone block reconstruction or remplissage may be more appropriate.^5^^,^^9^, ^10^, ^11^

A study by Lacheta et al.^8^ showed that the diameter of the “best-fit” circle differed significantly among 6 reviewers and represented a source of error when assessing GBL on both MRI and CT. Although several other studies have reported more favorable ICCs using this technique, their methods preclude the generalization of their findings to routine clinical practice.^17^^,^^18^ Sgroi et al.^18^ reported good to excellent reliability when assessing the ability of MRI to characterize bony Bankart lesions. However, their study was limited to 2 resident observers, and it is unclear whether readers were able to select their own sequences and images for measurement. Chalmers et al.^20^ showed good inter-rater reliability for GBL measurements from MRI; however, this was derived from measurements by 2 surgeons who were given preselected MRI slices from which to measure. Furthermore, the authors noted that despite ICCs within an acceptable range, the differences reported between the 2 observers would still have resulted in different treatment recommendations in over 30% of cases in their analysis. We evaluated the entirety of the perfect circle technique, which encompasses both the selection of the most representative MRI slice and the actual measurements, but we did not differentiate whether the poor reliability stemmed from the MRI slice or measuring. Simply put, measurements affecting surgical planning should not vary by observer in such a high proportion of cases. These findings call attention to the need for more reliable methods of assessing GBL, and we echo Chalmers et al. in calling for further research into alternative methods for assessing GBL.

The poor inter-rater reliability shown in this analysis is also of significance regarding interpreting the existing literature. Many existing studies reporting on operative techniques for the management of instability rely on the perfect circle technique for patient selection. However, if surgeons do not reliably measure the same degree of GBL, the ability to generalize these findings becomes limited. Additionally, the poor reliability of glenoid diameter measurement observed has additional clinical implications. For example, accurately calculating the glenoid track is dependent on the ability to correctly estimate both GBL and glenoid diameter using the perfect circle method.^21^^,^^22^ If our findings are representative of general trends among surgeons, the external validity of studies investigating the track concept may be called into question. This is further compounded by the poor inter-rater reliability for anterior GBL observed in this study. Together, these findings suggest that surgeons may be at risk of incorrectly classifying on- or off-track lesions using the perfect circle methodology.

### Limitations

Regarding limitations, we cannot evaluate the accuracy of measurements in this study because we did not have gold-standard 3-dimensionally reconstructed CT imaging with which to compare observer measurements. Additionally, the small numbers of cases and surgeons participating in this study represent a source of inherent bias in our analysis. A power analysis should have been performed to establish an appropriate sample size. We did not account for differing ICCs through various levels of GBL because greater GBL may produce less reliable measurements. This study did not identify what a significant difference between measurements clinically represented because all patients underwent the same procedure. Future studies should evaluate the clinical consequences these differences represent in a surgeon’s practice. Finally, because all measurements were obtained from 3-T MRI, we cannot extrapolate our findings to measurements taken from CT or 1.5-T MRI.

## Conclusions

The perfect circle technique for estimating anterior GBL on MRI was found to have moderate intrarater reliability; however, reliability between observers was found to be moderate to poor.

## Disclosure

All authors (N.P., C.K.G., E.I.W., A.G., K.F., K.J.K., R.H.R., J.P.S.) declare that they have no known competing financial interests or personal relationships that could have appeared to influence the work reported in this paper.

## Supplementary Data

ICMJE author disclosure forms
