# Supplementary material for: The Perfect Circle Technique Shows Poor Inter-rater Reliability in Measuring Anterior Glenoid Bone Loss on Magnetic Resonance Imaging
Source: Arthrosc Sports Med Rehabil. 2024 Feb 3;6(2):100905. doi: 10.1016/j.asmr.2024.100905 (PMC10901848; doi:10.1016/j.asmr.2024.100905)
Supplement: ICMJE author disclosure forms [file mmc1.docx]

Dr. Nata Parnes MD:

**Declaration of interests**
 
☒ The authors declare that they have no known competing financial interests or personal relationships that could have appeared to influence the work reported in this paper.
 
☐ The authors declare the following financial interests/personal relationships which may be considered as potential competing interests:

Clare Green:

**Declaration of interests**
 
☒ The authors declare that they have no known competing financial interests or personal relationships that could have appeared to influence the work reported in this paper.
 
☐ The authors declare the following financial interests/personal relationships which may be considered as potential competing interests:

Dr. Emily I. Wynkoop MD:

**Declaration of interests**
 
☒ The authors declare that they have no known competing financial interests or personal relationships that could have appeared to influence the work reported in this paper.
 
☐ The authors declare the following financial interests/personal relationships which may be considered as potential competing interests:

Dr. Adam Goldman DO:

**Declaration of interests**
 
☒ The authors declare that they have no known competing financial interests or personal relationships that could have appeared to influence the work reported in this paper.
 
☐ The authors declare the following financial interests/personal relationships which may be considered as potential competing interests:

Dr. Keith Fishbeck DO:

**Declaration of interests**
 
☒ The authors declare that they have no known competing financial interests or personal relationships that could have appeared to influence the work reported in this paper.
 
☐ The authors declare the following financial interests/personal relationships which may be considered as potential competing interests:

Dr. Kyle J. Klahs DO:

**Declaration of interests**
 
☒ The authors declare that they have no known competing financial interests or personal relationships that could have appeared to influence the work reported in this paper.
 
☐ The authors declare the following financial interests/personal relationships which may be considered as potential competing interests:

Dr. Robert H. Rolf MD:

**Declaration of interests**
 
☒ The authors declare that they have no known competing financial interests or personal relationships that could have appeared to influence the work reported in this paper.
 
☐ The authors declare the following financial interests/personal relationships which may be considered as potential competing interests:

Dr. John P. Scanaliato MD:

**Declaration of interests**
 
☒ The authors declare that they have no known competing financial interests or personal relationships that could have appeared to influence the work reported in this paper.
 
☐ The authors declare the following financial interests/personal relationships which may be considered as potential competing interests:

 
 
 
