# Supplementary material for: The Perfect Circle Technique Shows Poor Inter-rater Reliability in Measuring Anterior Glenoid Bone Loss on Magnetic Resonance Imaging
Source: Arthrosc Sports Med Rehabil. 2024 Feb 3;6(2):100905. doi: 10.1016/j.asmr.2024.100905 (PMC10901848; doi:10.1016/j.asmr.2024.100905)
Supplement: Appendix Table 1 [file mmc2.docx]

**Appendix Table 1.** Reviewer Measurements

| **Patient No.** | **Reviewer No.** | **Expected Diameter, mm** | **Glenoid Bone Loss, mm** | **% Glenoid Bone Loss** |
| --- | --- | --- | --- | --- |
| Patient 1 | 1 | 23.3 | 4.1 | 17.6 |
|  | 1 | 29.9 | 3.8 | 12.7 |
|  | 2 | 24.3 | 3.4 | 14.0 |
|  | 2 | 25.6 | 3.2 | 12.5 |
|  | 3 | 25.9 | 4.9 | 18.9 |
|  | 3 | 29.8 | 5.9 | 19.8 |
|  | 4 | 27.4 | 7.9 | 28.8 |
|  | 4 | 28.1 | 8 | 28.5 |
|  | 5 | 25.3 | 2.5 | 9.9 |
|  | 5 | 24.4 | 3.9 | 16.0 |
|  | 6 | 28.1 | 4.9 | 17.4 |
|  | 6 | 25.5 | 3.5 | 13.7 |
|  | 7 | 24.7 | 4 | 16.2 |
|  | 7 | 31.4 | 5.9 | 18.8 |
|  | 8 | 27.4 | 4.2 | 15.3 |
|  | 8 | 26.6 | 4 | 15.0 |
|  | 9 | 22.8 | 0 | 0.0 |
|  | 9 | 26 | 1.7 | 6.5 |
| Patient 2 | 1 | 27.2 | 5.1 | 18.8 |
|  | 1 | 27.7 | 3.5 | 12.6 |
|  | 2 | 22.9 | 3.2 | 14.0 |
|  | 2 | 28.2 | 5 | 17.7 |
|  | 3 | 23.4 | 2 | 8.5 |
|  | 3 | 27.8 | 0 | 0 |
|  | 4 | 27.6 | 6.3 | 22.8 |
|  | 4 | 27 | 6.1 | 22.6 |
|  | 5 | 24 | 0 | 0.0 |
|  | 5 | 21.7 | 0 | 0.0 |
|  | 6 | 23.8 | 4.6 | 19.3 |
|  | 6 | 29.1 | 6.5 | 22.3 |
|  | 7 | 25.2 | 3.5 | 13.9 |
|  | 7 | 28.5 | 5 | 17.5 |
|  | 8 | 26.7 | 4.3 | 16.1 |
|  | 8 | 28.9 | 4.1 | 14.2 |
|  | 9 | 25.4 | 3.3 | 13.0 |
|  | 9 | 24.4 | 2.8 | 11.5 |
| Patient 3 | 1 | 29.3 | 0 | 0 |
|  | 1 | 33.7 | 0 | 0 |
|  | 2 | 30.9 | 6.9 | 22.3 |
|  | 2 | 31.2 | 2 | 6.4 |
|  | 3 | 29.5 | 2.4 | 8.1 |
|  | 3 | 31.2 | 0 | 0 |
|  | 4 | 30.3 | 2.5 | 8.3 |
|  | 4 | 30.7 | 2.8 | 9.1 |
|  | 5 | 31.8 | 2.1 | 6.6 |
|  | 5 | 29.4 | 0 | 0.0 |
|  | 6 | 31.7 | 2.5 | 7.9 |
|  | 6 | 32.6 | 3.2 | 9.8 |
|  | 7 | 31.4 | 0 | 0.0 |
|  | 7 | 33.2 | 1.6 | 4.8 |
|  | 8 | 30.1 | 2.3 | 7.6 |
|  | 8 | 33 | 1 | 3.0 |
|  | 9 | 30.4 | 0 | 0.0 |
|  | 9 | 33.1 | 2.4 | 7.3 |
| Patient 4 | 1 | 27 | 3.8 | 14.1 |
|  | 1 | 30.3 | 3.4 | 11.2 |
|  | 2 | 27.4 | 2.3 | 8.4 |
|  | 2 | 29.3 | 2.5 | 8.5 |
|  | 3 | 24.9 | 0 | 0 |
|  | 3 | 28 | 0 | 0 |
|  | 4 | 28.5 | 3.6 | 12.6 |
|  | 4 | 28.3 | 3.5 | 12.4 |
|  | 5 | 21.7 | 0.8 | 3.7 |
|  | 5 | 24.7 | 0 | 0.0 |
|  | 6 | 33 | 2.9 | 8.8 |
|  | 6 | 34.3 | 2.9 | 8.5 |
|  | 7 | 27.9 | 1.3 | 4.7 |
|  | 7 | 28.9 | 2.1 | 7.3 |
|  | 8 | 27.1 | 3.2 | 11.8 |
|  | 8 | 27.6 | 3.4 | 12.3 |
|  | 9 | 24.7 | 1 | 4.0 |
|  | 9 | 27.6 | 0 | 0.0 |
| Patient 5 | 1 | 25.7 | 2.7 | 10.5 |
|  | 1 | 27.5 | 0 | 0 |
|  | 2 | 28.2 | 1.3 | 4.6 |
|  | 2 | 26.6 | 0.4 | 1.5 |
|  | 3 | 24.9 | 2 | 8 |
|  | 3 | 25.7 | 0 | 0 |
|  | 4 | 24.4 | 3 | 12.3 |
|  | 4 | 24 | 3 | 12.5 |
|  | 5 | 22.7 | 0 | 0.0 |
|  | 5 | 23.9 | 0 | 0.0 |
|  | 6 | 30.5 | 2.5 | 8.2 |
|  | 6 | 29.4 | 0.3 | 1.0 |
|  | 7 | 26.6 | 0 | 0.0 |
|  | 7 | 28.8 | 2.1 | 7.3 |
|  | 8 | 24.8 | 2.2 | 8.9 |
|  | 8 | 25.8 | 2.3 | 8.9 |
|  | 9 | 26.8 | 2.2 | 8.2 |
|  | 9 | 26 | 0 | 0.0 |
| Patient 6 | 1 | 25.8 | 0 | 0 |
|  | 1 | 28.7 | 3.6 | 12.5 |
|  | 2 | 27.6 | 1.5 | 5.4 |
|  | 2 | 29 | 1.3 | 4.5 |
|  | 3 | 26.4 | 3.7 | 14 |
|  | 3 | 25 | 2.3 | 9.2 |
|  | 4 | 26.4 | 3.6 | 13.6 |
|  | 4 | 27 | 3.8 | 14.1 |
|  | 5 | 26.8 | 0 | 0.0 |
|  | 5 | 26.5 | 1.9 | 7.2 |
|  | 6 | 29.7 | 1.8 | 6.1 |
|  | 6 | 31.4 | 3 | 9.6 |
|  | 7 | 32.2 | 2.7 | 8.4 |
|  | 7 | 30 | 2 | 6.7 |
|  | 8 | 27 | 3.6 | 13.3 |
|  | 8 | 29.4 | 3.7 | 12.6 |
|  | 9 | 27 | 0.8 | 3.0 |
|  | 9 | 28.7 | 0 | 0.0 |
| Patient 7 | 1 | 30.2 | 3.9 | 12.9 |
|  | 1 | 30.4 | 1.8 | 5.9 |
|  | 2 | 33.3 | 2.5 | 7.5 |
|  | 2 | 33.4 | 2.7 | 8.1 |
|  | 3 | 27.7 | 0 | 0 |
|  | 3 | 30.7 | 2.1 | 6.8 |
|  | 4 | 31.9 | 7.5 | 23.5 |
|  | 4 | 31.2 | 7.2 | 23.1 |
|  | 5 | 31.9 | 0 | 0.0 |
|  | 5 | 29.6 | 0 | 0.0 |
|  | 6 | 31.7 | 2.6 | 8.2 |
|  | 6 | 33.9 | 1.9 | 5.6 |
|  | 7 | 33.1 | 2.2 | 6.6 |
|  | 7 | 34.2 | 3 | 8.8 |
|  | 8 | 29.8 | 1.5 | 5.0 |
|  | 8 | 31 | 1.8 | 5.8 |
|  | 9 | 29.4 | 0 | 0.0 |
|  | 9 | 31.9 | 2.7 | 8.5 |
| Patient 8 | 1 | 29 | 3.8 | 13.1 |
|  | 1 | 29.8 | 2.8 | 9.4 |
|  | 2 | 33.9 | 4.1 | 12.1 |
|  | 2 | 33.9 | 2.8 | 8.3 |
|  | 3 | 29.6 | 2.9 | 9.8 |
|  | 3 | 30.5 | 4.1 | 13.4 |
|  | 4 | 28.3 | 3.5 | 12.4 |
|  | 4 | 29 | 3.9 | 13.4 |
|  | 5 | 27.7 | 1.8 | 6.5 |
|  | 5 | 26 | 1 | 3.8 |
|  | 6 | 32.2 | 1.1 | 3.4 |
|  | 6 | 35.5 | 2.1 | 5.9 |
|  | 7 | 31.8 | 2.6 | 8.2 |
|  | 7 | 32.1 | 2.5 | 7.8 |
|  | 8 | 29.1 | 2.7 | 9.3 |
|  | 8 | 31 | 1.8 | 5.8 |
|  | 9 | 31.9 | 0 | 0.0 |
|  | 9 | 28.9 | 0.9 | 3.1 |
| Patient 9 | 1 | 29.6 | 0 | 0 |
|  | 1 | 31.3 | 3.2 | 10.2 |
|  | 2 | 32.4 | 4.1 | 12.7 |
|  | 2 | 32.3 | 2.3 | 7.1 |
|  | 3 | 30.9 | 3.6 | 11.7 |
|  | 3 | 27.6 | 0 | 0 |
|  | 4 | 30.8 | 6.1 | 19.8 |
|  | 4 | 31.4 | 6.8 | 21.7 |
|  | 5 | 29.9 | 0 | 0.0 |
|  | 5 | 28.6 | 0 | 0.0 |
|  | 6 | 34.1 | 2.3 | 6.7 |
|  | 6 | 38.1 | 3.6 | 9.4 |
|  | 7 | 32.6 | 2.8 | 8.6 |
|  | 7 | 32.6 | 3 | 9.2 |
|  | 8 | 29.7 | 1.9 | 6.4 |
|  | 8 | 28 | 2.1 | 7.5 |
|  | 9 | 31.5 | 3.5 | 11.1 |
|  | 9 | 31.4 | 0 | 0.0 |
| Patient 10 | 1 | 29.7 | 3.2 | 10.8 |
|  | 1 | 34.6 | 2.6 | 7.5 |
|  | 2 | 28 | 5 | 17.9 |
|  | 2 | 28.9 | 5.2 | 18.0 |
|  | 3 | 26.4 | 0 | 0 |
|  | 3 | 30.1 | 0 | 0 |
|  | 4 | 26.6 | 6.4 | 24.1 |
|  | 4 | 25.7 | 6 | 23.3 |
|  | 5 | 29.6 | 0 | 0.0 |
|  | 5 | 26.8 | 0 | 0.0 |
|  | 6 | 33.8 | 1.9 | 5.6 |
|  | 6 | 31.2 | 0 | 0.0 |
|  | 7 | 28.7 | 0 | 0.0 |
|  | 7 | 29.8 | 1.3 | 4.4 |
|  | 8 | 28.7 | 2 | 7.0 |
|  | 8 | 30 | 1.7 | 5.7 |
|  | 9 | 26.6 | 0 | 0.0 |
|  | 9 | 27.2 | 0 | 0.0 |
